# A Novel L-ascorbate Peroxidase 6 Gene, *ScAPX6*, Plays an Important Role in the Regulation of Response to Biotic and Abiotic Stresses in Sugarcane

**DOI:** 10.3389/fpls.2017.02262

**Published:** 2018-01-17

**Authors:** Feng Liu, Ning Huang, Ling Wang, Hui Ling, Tingting Sun, Waqar Ahmad, Khushi Muhammad, Jinxin Guo, Liping Xu, Shiwu Gao, Youxiong Que, Yachun Su

**Affiliations:** ^1^Key Laboratory of Sugarcane Biology and Genetic Breeding, Ministry of Agriculture, Fujian Agriculture and Forestry University, Fuzhou, China; ^2^Department of Genetics, Hazara University, Mansehra, Pakistan; ^3^Guangxi Collaborative Innovation Center of Sugarcane Industry, Guangxi University, Nanning, China

**Keywords:** sugarcane, L-ascorbate peroxidase 6 gene, subcellular localization, biotic and abiotic stresses, transient overexpression

## Abstract

The L-ascorbate peroxidase 6 gene (*APX6*) is one of the most important genes for scavenging H_2_O_2_ and plays a vital role in plant resistance to environmental stresses. In this study, a novel *ScAPX6* gene (GenBank Accession No. KT907352) was obtained from a sugarcane variety (ROC22). Bioinformatics analysis showed that ScAPX6 has a cDNA length of 1,086 bp and encoded 333 amino acid residues. Subcellular localization confirmed that ScAPX6 was located in the chloroplast. Enhanced growth of *Escherichia coli* BL21 cells that expressed ScAPX6 showed high tolerance under copper (Cu) stress. Real-time quantitative PCR analysis revealed that *ScAPX6* was constitutively expressed wherein with the highest expression levels in sugarcane pith and leaf and the lowest in the root. *ScAPX6* was down-regulated by salicylic acid (SA), hydrogen peroxide (H_2_O_2_), polyethylene glycol (PEG) and sodium chloride (NaCl) stimuli. Interestingly, it was significantly up-regulated under the stresses of abscisic acid (ABA) and methyl jasmonate (MeJA) wherein with the highest inducible expression levels at 6 h at 6.0- and 70.0-times higher, respectively than that of control. Overexpression of *ScAPX6* in *Nicotiana benthamiana* leaves enhanced the resistance to the infection of tobacco pathogens *Pseudomonas solanacearum* and *Fusarium solani* var. *coeruleum*. These results implied that *ScAPX6* might positively respond to ABA, MeJA, and Cu, but might negatively respond to the stresses of SA, H_2_O_2_, PEG, and NaCl. Keeping in view the current investigation, *ScAPX6* could be associated with the hypersensitive response (HR) or immunity of sugarcane, which will provide a baseline for the function identification of sugarcane *ScAPX6*.

## Introduction

In addition to *Oryza sativa, Triticum aestivum*, and *Zea mays*, sugarcane is the fourth largest staple food for the people of China. Sugarcane planting and production are of great significance in sugar supply (Li, 2000). However, the growth and development of sugarcane is severely affected by various abiotic and biotic stresses, such as drought, cold, salinity, heavy metals, high temperature, viruses, fungi, and so on (Li, 2000; Xu et al., 2008). As reported, environmental stimuli can induce active oxygen system which may cause injury to plant cells (Mittler et al., 2004, 2011). Peroxidases (EC number 1.11.1.x), including glutathione peroxidase (GPX), catalase (CAT), and ascorbate peroxidase (APX), are widespread in organisms and can remove the reactive oxygen (Shigeoka et al., 2002; Apel and Hirt, 2014). APX, belonging to type I heme peroxidase and copper oxidase family, is widely employed in plants and can rapidly scavenge hydrogen peroxide (H_2_O_2_) in the ascorbic acid (ASA) and glutathione (GSH) cycle (Shigeoka et al., 2002). The role of APX is highly specific to ascorbic acid, that is, to help electron donor to oxidation (Chen and Asada, 1989; Mittler, 2002; Foyer and Noctor, 2005).

According to the orientation characteristic, there are three mainly APX subfamilies in plants, such as cytoplasm APX (cAPX), thylakoid APX (tAPX), and APx-R (Apx-Related) (Mano et al., 1997; Shigeoka et al., 2002; Chew et al., 2003; Dunand et al., 2011). *APXs* gene have been reported in several plants such as *Nelumbo nucifera* (Chen et al., 2011), *Hordeum vulgare* (Shi et al., 2001), *Solanum tuberosum* (Kawakami et al., 2002), *Z. mays* (Breusegem et al., 1995), and *Vitis pseudoreticulata* (Lin et al., 2006). The expression of *APX* can be regulated by environmental stimuli, such as salt (Badawi et al., 2004), temperature (Kawakami et al., 2002), high light (Maruta et al., 2010), and heavy metal stresses (Pallavi and Dubey, 2007). Research has also shown that *APX* induced by adversity stress can regulate the content of H_2_O_2_ in the cell and redox signaling, and then affect plant tolerance to the osmotic stress (Andréia et al., 2012). This characteristic of APX enzyme activity may be treated as one of the physiological and biochemical indexes measuring crop resistance to biotic and abiotic stresses, for instance, water deficit (Nayyar and Gupta, 2006) and high temperature (Almeselmani et al., 2006). Kornyeyev et al. (2001) transferred the chloroplast *APX* gene into *Gossypium* spp., and it was found that APX activity in the transgenic cotton leaf was higher than that of the wild type. Overexpression of *tAPX* genes increased the resistance of *Nicotiana tabacum* and *Arabidopsis thaliana* to the oxidative stress induced by methyl violet essence (Yabuta et al., 2002; Murgia et al., 2004).

Until now, there are four nucleotide sequences of *APX* genes, which have been identified in sugarcane. Wang Z. Q. et al. (2015) indicated that the APX enzyme activity in sugarcane smut resistant variety Yacheng05-179 was significantly higher than the susceptible variety Liucheng03-182 after inoculated with *Sporisorium scitamineum*. As a result the expression level of one sugarcane *ScAPX* gene (GenBank Acc. No. KJ7565501) increased under the stresses of salicylic acid (SA), methyl jasmonate (MeJA), abscisic acid (ABA), H_2_O_2_, sodium chloride (NaCl), and polyethylene glycol (PEG). Wang S. et al. (2015) demonstrated that *TAPX* gene (GenBank Acc. No. JQ958327) played a part in sugarcane resistance to osmotic stress. Huang et al. (2013) found that sugarcane *S*-*APX2* gene showed highly homologous with rice *APX* (GenBank Acc. No. XP_002463451.1) and mazie *APX* (GenBank Acc. No. DAA41857.1). Another sugarcane *APX* gene (GenBank Acc. No. KX235995) was found in *Saccharum arundinaceum*, but its function was unclear.

From all the above, cloning the *APXs* gene of different isozymes is necessary to better understand the *APX* gene family and know more about their expression levels under different stress conditions. In the present study, a putative *APX6* unigene, named as *ScAPX6*, was cloned and identified based on our previous transcriptome data of sugarcane in response to sorghum mosaic virus (SrMV) infection (Bioproject number: PRJNA379719). The sequence characters of *ScAPX6* was analyzed by bioinformatics analysis, and the expression patterns of *ScAPX6* gene after exposure to various stresses, such as ABA, MeJA, SA, H_2_O_2_, PEG, NaCl, and copper (Cu), were detected by real-time quantitative polymerase chain reaction (qRT-PCR). Furthermore, its expression in *Escherichia coli*, subcellular localization and transient overexpression in *Nicotiana benthamiana* were also investigated. This study will be helpful to understand the gene function of *ScAPX6* in sugarcane.

## Materials and methods

### Plant material and treatments

For the analysis of the tissue-specific expression of *ScAPX6*, six healthy plants of 10 months old of ROC22 were used, and then +1 leaf, root, bud, skin and pith were collected. The samples were fixed in liquid nitrogen and stored at −80°C until the extraction of total RNA.

For the abiotic treatment, uniform tissue cultured plantlets of ROC22 at the five or six leaf stage were transferred to water for one week and then treated by the following six stress conditions with root dipping of 5 mmol·L^−1^ SA, 100 μmol·L^−1^ MeJA, 100 μmol·L^−1^ ABA, 10 μmol·L^−1^ H_2_O_2_, 25.0% PEG 8000, 250 mmol·L^−1^ NaCl, and 100 mmol·L^−1^ copper chloride (CuCl_2_), at 28°C with 16 h light and 8 h darkness (Su et al., 2014a). The whole plantlets under SA, MeJA, H_2_O_2_, PEG, and NaCl stresses were harvested at 0, 6, 12, and 24 h, respectively. Another set of plantlets under Cu stress was harvested at 0, 12, 24, and 48 h, respectively. Three plantlets per time point were gathered and immediately fixed in liquid nitrogen, and stored at −80°C until the extraction of total RNA.

### Total RNA extraction and the first-strand cDNA synthesis

Total RNA of the treated samples was extracted by Trizol® Reagent (Invitrogen, Carlsbad, CA, USA) according to the manufacturer's instructions. The first-strand cDNA synthesis was performed using Prime-Script^TM^ RT Reagent Kit (TaKaRa, Dalian, China) following manufacturer's instructions and tested by 1% agarose gel electrophoresis.

### Sugarcane *ScAPX6* gene isolation and gateway entry vector construction

The sequence of a putative *APX6* unigene (*ScAPX6*) from our previous transcriptome data of sugarcane in response to SrMV infection was used to design the cloning primer APX6-1F/1R (Table 1). The first-strand cDNA of ROC22 was used as amplification template. The reverse transcription–polymerase chain reaction (RT-PCR) procedure was 94°C for 4 min; 94°C for 30 s, 55°C for 30 s, 72°C for 2 min, 35 cycles; and 72°C for 10 min. RT-PCR products were gel-purified and cloned into pMD19-T vector (TaKaRa, Dalian, China), and then transformed into *E. coli* DH5α competent cells and sequenced (Sangon, Shanghai, China).

**Table 1:** Primers used in this study.

**Primer**	**Sequence information (5′–3′)**	**Strategy**
APX6-1F	CTTGAGAAGGCAAGCCAGGA	Gene cloning
APX6-1R	CGAGACACTGGTACAGGGGA	Gene cloning
APX6-2F	GATTTGATTGCCGTGGCTGG	qRT-PCR analysis
APX6-2R	TCTTCAGGAAGTTTGCCAGTTG	qRT-PCR analysis
CUL-F	TGCTGAATGTGTTGAGCAGC	qRT-PCR analysis
CUL-R	TTGTCGCGCTCCAAGTAGTC	qRT-PCR analysis
CAC-F	ACAACGTCAGGCAAAGCAAA	qRT-PCR analysis
CAC-R	AGATCAACTCCACCTCTGCG	qRT-PCR analysis
APX6-3F	GGGGACAAGTTTGTACAAAAAAGCAGGCTTCATGGAGCTCACCAACATCCC	Gateway entry vector construction and RT-PCR analysis
APX6-3R	GGGGACCACTTTGTACAAGAAAGCTGGGTCAGCTGTTCTCCACGAGGCTC	Gateway entry vector construction and RT-PCR analysis
APX6-4F	CAGTGGTCTCACAACATGGAGCTCACCAACATCCC	Subcellular localization vector construction
APX6-4R	CAGTGGTCTCATACAAGCTGTTCTCCACGAGGCTC	Subcellular localization vector construction
NtHSR201-F	CAGCAGTCCTTTGGCGTTGTC	qRT-PCR analysis
NtHSR201-R	GCTCAGTTTAGCCGCAGTTGTG	qRT-PCR analysis
NtHSR203-F	TGGCTCAACGATTACGCA	qRT-PCR analysis
NtHSR203-R	GCACGAAACCTGGATGG	qRT-PCR analysis
NtHSR515-F	TTGGGCAGAATAGATGGGTA	qRT-PCR analysis
NtHSR515-R	TTTGGTGAAAGTCTTGGCTC	qRT-PCR analysis
NtPR-1a/c-F	AACCTTTGACCTGGGACGAC	qRT-PCR analysis
NtPR-1a/c-R	GCACATCCAACACGAACCGA	qRT-PCR analysis
NtPR2-F	TGATGCCCTTTTGGATTCTATG	qRT-PCR analysis
NtPR2-R	AGTTCCTGCCCCGCTTT	qRT-PCR analysis
NtPR3-F	CAGGAGGGTATTGCTTTGTTAGG	qRT-PCR analysis
NtPR3-R	CGTGGGAAGATGGCTTGTTGTC	qRT-PCR analysis
NtEFE26-F	CGGACGCTGGTGGCATAAT	qRT-PCR analysis
NtEFE26-R	CAACAAGAGCTGGTGCTGGATA	qRT-PCR analysis
NtAccdeaminase-F	TCTGAGGTTACTGATTTGGATTGG	qRT-PCR analysis
NtAccdeaminase-R	TGGACATGGTGGATAGTTGCT	qRT-PCR analysis
NtEF1-α-F	TGCTGCTGTAACAAGATGGATGC	qRT-PCR analysis and RT-PCR analysis
NtEF1-α-R	GAGATGGGGACAAAGGGGATT	qRT-PCR analysis and RT-PCR analysis

*attB1 and attB2 adapters were underlined in the forward primer APX6–3F and in the reverse primer APX6–3R, respectively*.

The open reading frame (ORF) of *ScAPX6* with Gateway entry adapters attB1 and attB2 was amplified from the plasmid of pMD19-T-*ScAPX6* by the primers (APX6-3F/3R) (Table 1). The touchdown PCR procedure was 94°C for 4 min; 94°C for 30 s, 70°C for 30 s and then each loop drop 0.5°C, 72°C for 1 min and 30 s, 35 cycles; and 72°C for 10 min. The PCR amplification products were gel-purified and transformed into the Gateway^@^ donor vector of pDONR221 (Invitrogen, USA) following the manufacturer's instructions of Gateway® BP Clonase™ II Enzyme Mix (Invitrogen, USA). The mixture of BP reaction was transformed into DH5α competent cells and sequenced (Sangon, Shanghai, China). The positive plasmid pDONR221-*ScAPX6* was achieved and then used for the constructions of prokaryotic expression vector and eukaryotic expression vector.

### Bioinformatics analysis

The ORF was translated and analyzed by ORF Finder (https://www.ncbi.nlm.nih.gov/orffinder/). Conserved domain of ScAPX6 was predicted by the SMART program (http://smart.embl-heidelberg.de/) and NCBI Conserved Domains Database (CDD) (http://www.ncbi.nlm.nih.gov/Structure/cdd/wrpsb.cgi). The ExPASy tool (http://us.expasy.org/tools) was used to predict the basic physical and chemical properties of ScAPX6. The cleavage sites of the signal peptides were predicted by SignalP 4.1 Server (http://www.cbs.dtu.dk/services/SignalP/). Prediction of transmembrane helices in ScAPX6 protein was performed by TMHMM Server v. 2.0 (http://www.cbs.dtu.dk/services/TMHMM-2.0/). Psort software was used to predict the subcellular localization of ScAPX6. GOR IV software (https://npsa-prabi.ibcp.fr/cgi-bin/npsa_automat.pl?page=/NPSA/npsa_gor4.html) was used to analyze the secondary structure of ScAPX6. The protein 3D model was predicted by SWISSMODEL software (http://swissmodel.expasy.org/). The homologous sequences of ScAPX6 were obtained using Blastp in NCBI. DNAMAN software was used for the multiple sequence alignment. The phylogenetic tree of ScAPX6 was constructed with amino acid sequences from other species by the neighbor-joining (NJ) method (1,000 bootstrap replicates) using the MEGA 6.06 (Saitou and Nei, 1987).

### Subcellular colocalization assay

The ORF of *ScAPX6* was amplified by the primer APX6–4F/4R, and then was inserted into the *Bsa*I and *Eco*31I restriction sites of the pBWA(V)HS-ccdb-GLosgfp vector. Then the recombinant vector pBWA(V)HS-*ScAPX6*-GLosgfp and the chloroplast marker vector were co-transformed in rice protoplasts with PEG solution (40% W/V PEG 4000, 0.2 mol·L^−1^ mannitol and 0.1 mol·L^−1^ calcium chloride). The mixture was cultured in dark for 30 min, and then the protoplasts was gathered and cultured in dark for 16–24 h. The method of transformation of rice protoplasts was followed by Datta and Datta (1999). The subcellular localization of the fusion protein was observed by a confocal laser scanning microscope Leica TCS SP5 (Germany).

### Expression of *ScAPX6* in *E. coli* BL21 (DE3) strain

The plasmid of pDONR221-*ScAPX6* was digested with *Ava*I and then gel-purified for LR reaction with prokaryotic expressive vector of pEZYHb according to the manufacturer's instructions of LR Clonase™ II Enzyme Mix (Invitrogen, USA). The recombinant plasmid of pEZYHb-ScAPX6 was transformed into the competent cells *E. coli* BL21 (DE3) and then induced by 1.0 mmol·L^−1^ isopropyl β–D-thiogalactoside (IPTG) at 28°C for 0, 2, 4, and 8 h (Guo et al., 2008). LB medium with *E. coli* BL21 (blank) and BL21+pEZYHb (control) were induced by 1.0 mmol·L^−1^ IPTG for 0 and 8 h, respectively. The collected bacterial protein was analyzed by 12% sodium dodecyl sulfate polyacrylamide gel electrophoresis (SDS-PAGE).

Spot assay was conducted to study the responses of *E. coli* BL21 cells expressing the *ScAPX6* gene under abiotic stress, such as NaCl, Cu and PEG. When OD_600_ of *E. coli* BL21 cells in LB medium (containing 80 μg·mL^−1^ ampicillin) reached to 0.6, IPTG with a concentration of 1.0 mmol·L^−1^ was added, and then the cells were grown at 37°C for 12 h. The cultures were diluted to OD_600_ = 0.6, and then diluted to two levels of 10^−3^ and 10^−4^ (Guo et al., 2012). Ten microliters from each level was spotted on LB plates containing NaCl (250, 500, and 750 mmol·L^−1^), CuCl_2_ (250, 500, and 750 μmol·L^−1^) and PEG (15, 30, and 45%), respectively (Su et al., 2013). All plates were cultured in 37°C overnight and photographed.

### Gene expression patterns of *ScAPX6*

SYBR Green Master (ROX) (Roche, China) and a 7500 qRT-PCR system (Applied Biosystems, South San Francisco, CA, USA) were applied to analyze gene expression levels of *ScAPX6* in different tissues and in response to various stresses. The sequence-specific primer of *ScAPX6* (APX6-2F/2R) (Table 1) was designed by Premier 5.0 software. The primer combination of clathrin adaptor complex (*CAC*) and cullin (*CUL*) (Table 1) was regarded as the internal control (Guo et al., 2014). The 20 μL reaction system containing 10 μL SYBR Green Master Mix, 0.8 μL each of 10 μmol·L^−1^ upstream and downstream primers, 2 μL cDNA templates (20 × diluted cDNA) and 6.4 μL double distilled water. Each qRT-PCR was conducted in triplicate. The qRT-PCR procedure was 50°C for 2 min; 95°C for 10 min; 35 cycles of 95°C for 15 s, and 60°C for 1 min. The 2^−ΔΔCt^ method (Livak and Schmittgen, 2001) was employed to analyze the qRT-PCR data.

### Transient overexpression of *ScAPX6* in *N. benthamiana*

To study the role of *ScAPX6* in response to pathogen infection and its hypersensitive reaction in plant, an overexpressed vector pEarleyGate 203-*ScAPX6* was constructed by Gateway cloning technique according to the manufacturer's instructions of LR Clonase™ II Enzyme Mix (Invitrogen, USA). *N. benthamiana* leaves was inoculated with the vector of pEarleyGate 203-*ScAPX6* by an *Agrobacterium*-mediated transient expression method conducted by Su et al. (2014b). Two important tobacco pathogens, *Pseudomonas solanacearum* and *Fusarium solani* var. *coeruleum*, were cultured in potato dextrose water (PDW) liquid medium at 28°C. When the two pathogens cells were cultured to an OD_600_ of 0.8, they were separately infected into the treated leaves that were agroinfiltrated with pEarleyGate 203-*ScAPX6* for 24 h. Then the 3,3′-diaminobenzidine (DAB) staining, trypan blue staining and transcript analysis of the eight tobacco immunity-associated marker genes (Table 1), were conducted by the treated *N. benthamiana* leaves according to Su et al. (2016). RT-PCR was used to detect whether *ScAPX6* has been overexpressed in *N. benthamiana*, with the RNA of treated leaves and *ScAPX6* specific primer (APX6-3F/3R, Table 1), the *NtEF1*-α was treated as control. RT-PCR procedure was 94°C for 4 min; 94°C for 30 s, 72°C for 30 s, 72°C for 2 min, 35 cycles; and 72°C for 10 min. All treatment materials were cultured at 24°C (16 h light/8 h darkness) and then photographed at 1 and 7 day (d) separately. Each test was repeated three times.

DAB and trypan blue staining were used for histochemical analysis of *Agrobacterium*-infiltrated leaves. The leaves was soaked in DAB-HCl solution (1.0 mg·mL^−1^, pH 5.8), and then cultured in the dark for 12 h. The leaves were destained with 95% ethanol at 100°C for 10 min (Su et al., 2014a), and then was imaged for H_2_O_2_ detection with a stereoscopic microscope (Nikon, Tokyo, Japan) and a light microscope (Leica, Wetzlar, Germany). Three biological replicates were prepared. The leaves were also deal with trypan blue mixture, containing 10 mL lactic acid, 10 g phenol, 10 mL glycerol, 30 mL absolute ethanol, 10 mg trypan blue, and 10 mL distilled water, and then was boiled for 5 min. After staining, the leaves were soaked in a chloral hydrate solution (2.5 g·mL^−1^) for decoloring (Dang et al., 2013). The blue color of the leaves for the cell death was also imaged with a stereoscopic microscope (Nikon, Tokyo, Japan) and a light microscope (Leica, Wetzlar, Germany).

## Results

### Cloning and bioinformatics analysis of ScAPX6

**Figure 1:**
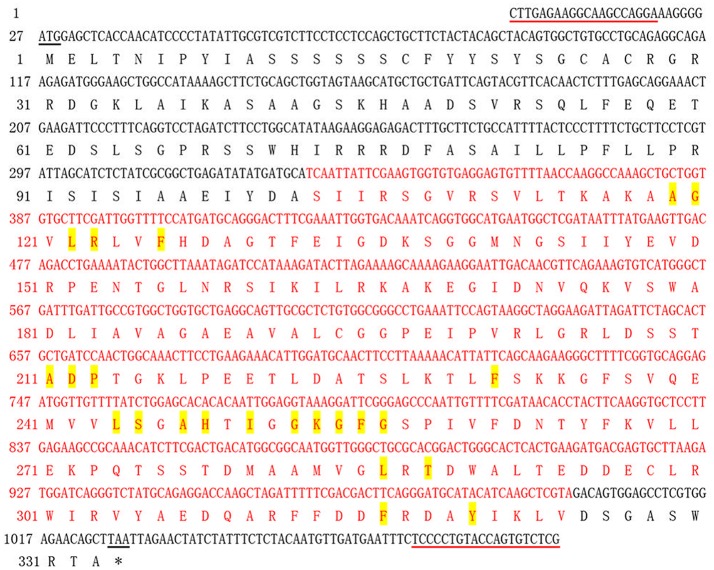
Nucleotide acid sequence and deduced amino acid sequence of sugarcane *ScAPX6* gene obtained by RT-PCR. The start codon and termination codon were underlined in black. The primer used in RT-PCR was underlined in red line. The peroxidase like superfamily domain contains 222 amino acids (from 103 to 324) was highlighted in red. The amino acids highlighted in yellow represented the heme binding site. ^*^, Stop codon.

In the present study, a full-length cDNA of *APX6* unigene, which was named as *ScAPX6* (GenBank Acc. No. KT907352), was isolated from ROC22. The cDNA sequence length of *ScAPX6* was 1,086 bp (Figure S1) with a complete ORF (1,002 bp, from position 27 to position 1,025), encoding 333 amino acid residues. ScAPX6 had a molecular mass of 36.21 kDa and an isoelectric point (*pI*) of 6.91. CDD search of NCBI showed that ScAPX6 belonged to a member of the plant-peroxidase-like superfamily (Figure 1). The instability index of ScAPX6 protein was 48.10, suggesting that ScAPX6 might be an unstable acid hydrophilic protein (Walker, 2005). Secondary structure prediction of ScAPX6 predicted that the percentages of alpha-helix, random coil, and extended strand were 44.74, 42.94, and 12.31%, respectively.

**Figure 2:**
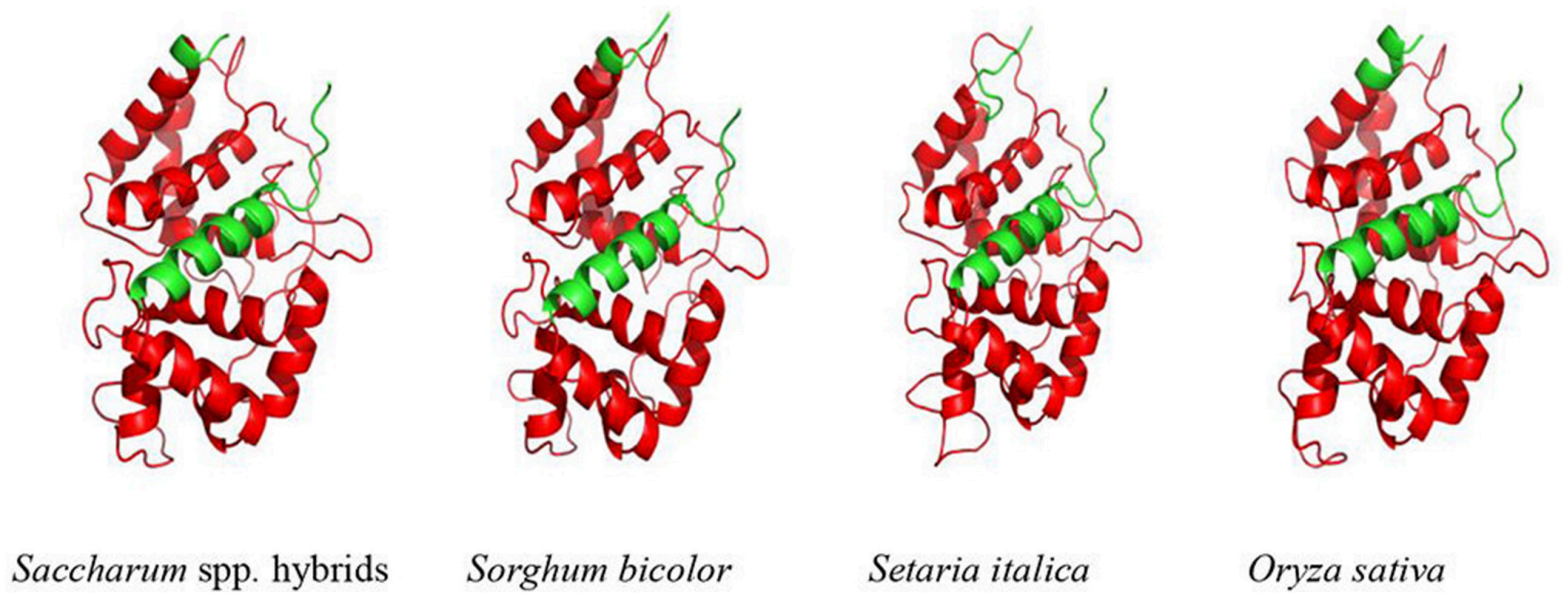
Predicted 3D structure of ScAPX6. The plant-peroxidase-like domain was in red. *Saccharum* spp. hybrids (AMQ80947.1), *Sorghum bicolor* (XP_002445876.1), *Setaria italica* (XP_004973913.1), *Oryza sativa* Japonica Group (EAZ43377.1).

Furthermore, SWISSMODEL program showed that the main spatial structures of ScAPX6 were alpha-helix and random coil (Figure 2). Comparing ScAPX6 with *O. sativa* Japonica Group APX6 (EAZ43377.1), *S. italic* APX6 (XP_004973913.1), and *S. bicolor* APX6 (XP_002445876.1), we found that the spatial structure of these four APX6 was basically in line with each other, suggesting that ScAPX6 owned high conservation of spatial structure with different plant species. Psort software predicted that ScAPX6 might be located in the chloroplast thylakoid membrane, plasma membrane, chloroplast stroma, and chloroplast thylakoid space with the probabilities of 71.9, 65.0, 56.1, and 56.1%, respectively.

**Figure 3:**
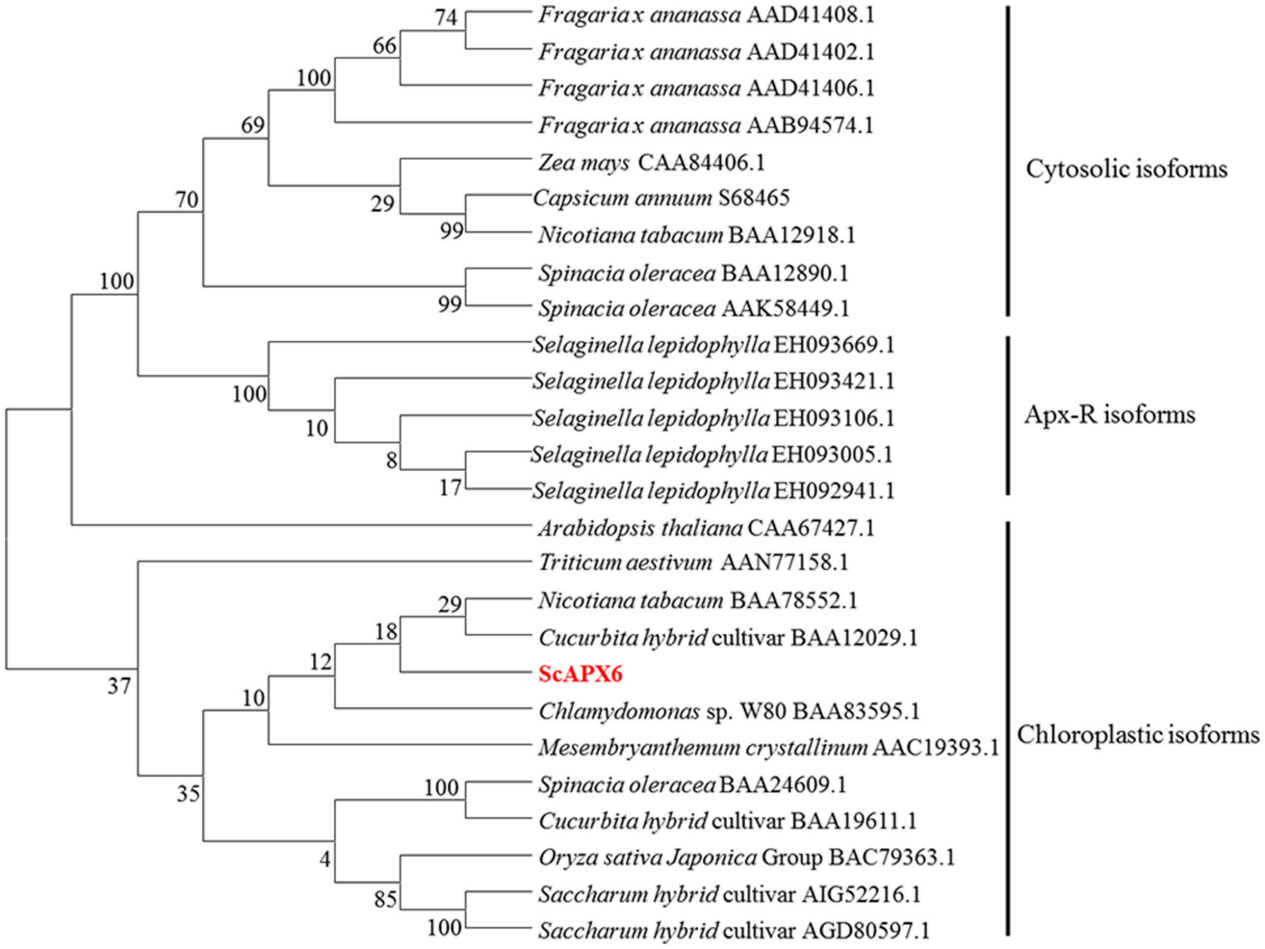
Phylogenetic analysis of deduced amino acid sequence from ScAPX6 and other ascorbate peroxidases proteins. The GenBank accession number of proteins were according to Teixeira et al. (2004) and downloaded from NCBI. The neighbor-joining method with 1,000 bootstrap replications was used.

According to the classification method by Teixeira et al. (2004), the phylogenetic tree was separated into three groups, including cytosolic isoforms, Apx-R isoforms and chloroplastic isforms (Figure 3). ScAPX6 was clustered into group chloroplastic isforms. Two APX proteins reported in *Saccharum* hybrid cultivar, which were ScAPX (AIG52216.1) and TAPX (AGD80596.1), were also clustered into the same clade as ScAPX6.

### Subcellular localization

**Figure 4:**
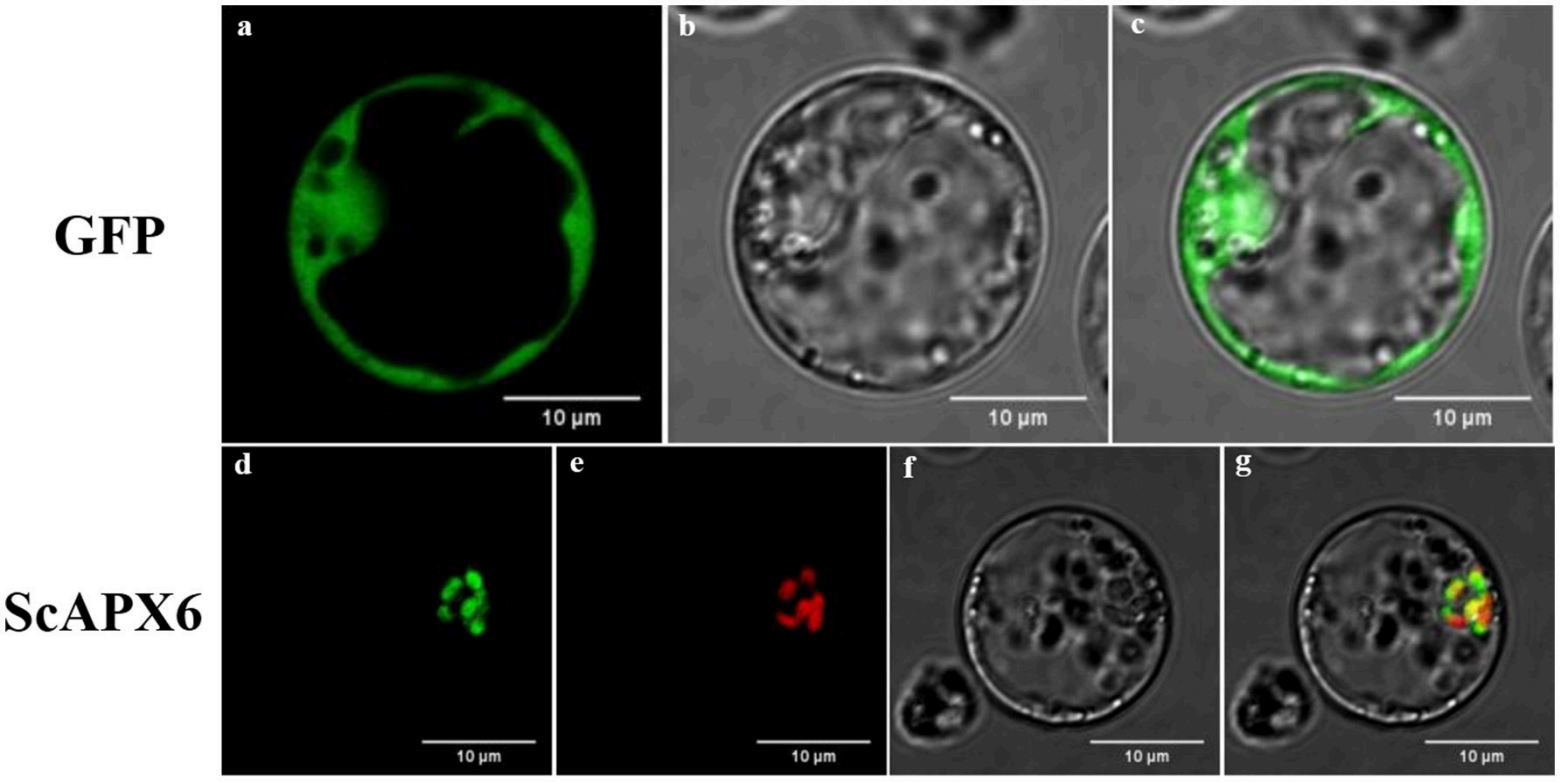
Subcellular localization analysis of ScAPX6 in rice protoplasts. **(a,d)** green fluorescence; **(b,f)** visible light; **(e)** red fluorescence from chloroplast marker; **(c,g)** merged light.

The recombinant vector pBWA(V)HS-ScAPX6-GLosgfp was constructed to understand the subcellular location of ScAPX6. The results showed that ScAPX6 and the chloroplast marker were located in the same place, so it was confirmed that ScAPX6 was located in the chloroplast, which is in accordance with the results of prediction (Figure 4).

### Expression of *ScAPX6* in *E. coli* BL21 (DE3) strain

**Figure 5:**
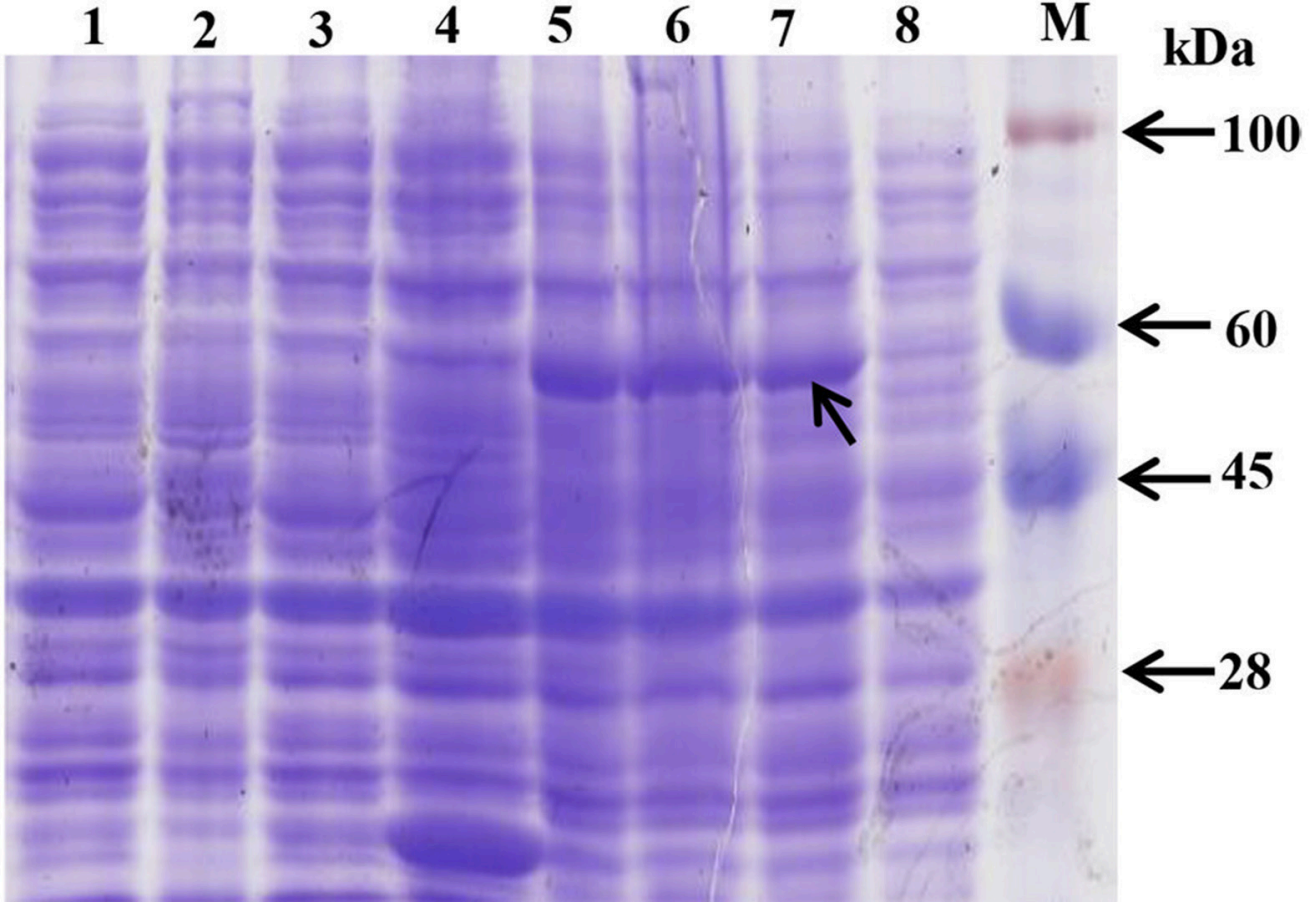
Prokaryotic expression of pEZY-Hb-ScAPX6 fusion protein in *Escherichia coli* BL21 (DE3). M, protein marker; 1, BL21 cell induction for 8 h; 2, BL21 cell without induction; 3, pEZY-Hb without induction; 4, pEZY-Hb induction for 8 h; 5–8, pEZY-Hb-*ScAPX6* induction for 8, 4, 2, and 0 h, respectively.

The *ScAPX6* gene was combined with the expression vector pEZYHb and then was transformed into *E. coli* BL21 cell. The SDS-PAGE analysis (Figure 5) showed that ScAPX6 was expressed as a recombinant protein in the BL21 cells. In Figure 5, after induced by 1.0 mmol·L^−1^ IPTG at 28°C for 2, 4, and 8 h, an obvious accumulation protein (including the 6× His-tag) at approximate 55 kDa was observed.

### Overexpression of *ScAPX6* in *E. coli* enhances cell growth under Cu stress

**Figure 6:**
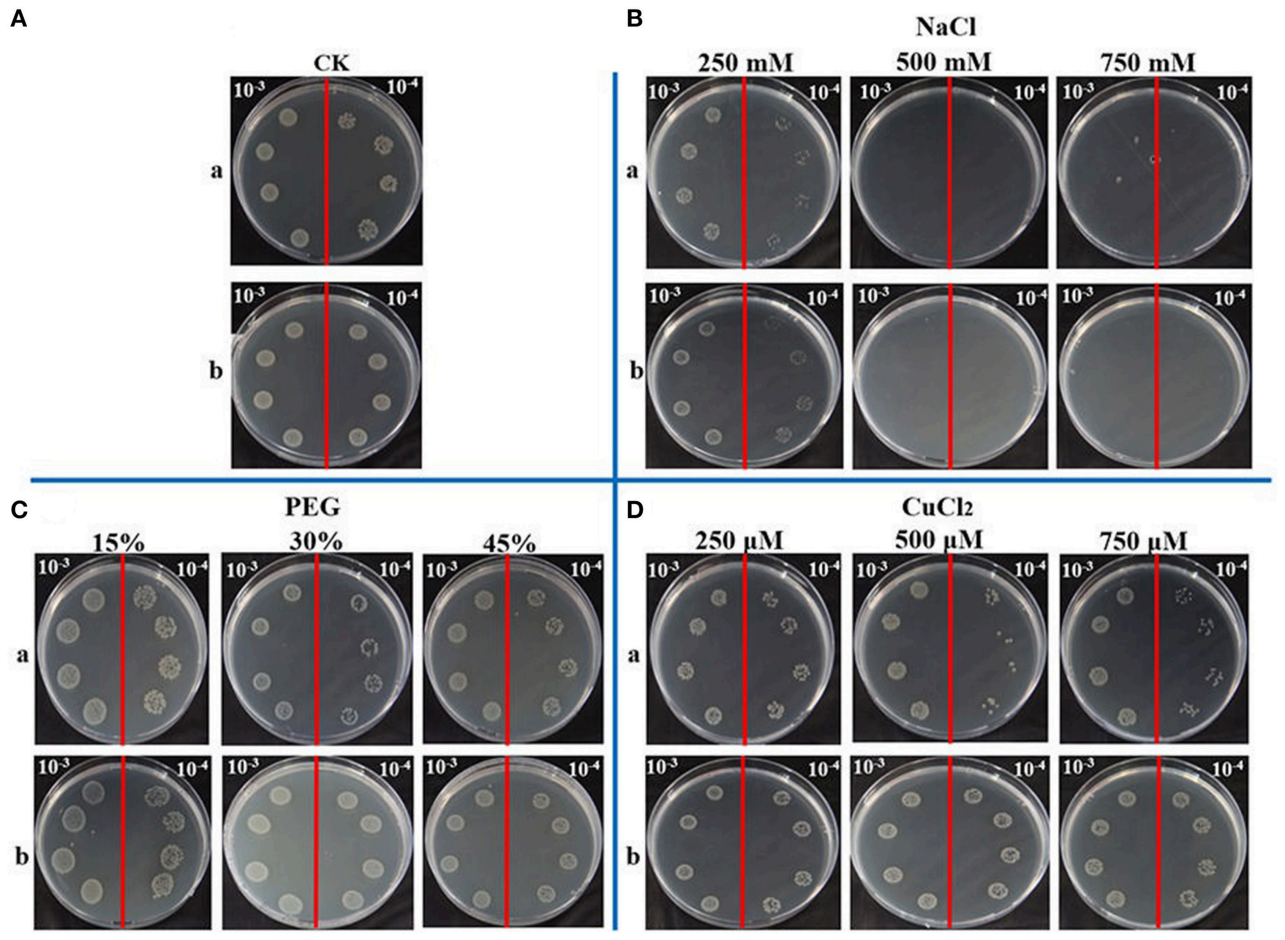
Spot assays of BL21+pEZY-Hb-ScAPX6 **(b)** and BL21+pEZY-Hb (control) **(a)** on LB plates with NaCl, PEG and CuCl_2_. Isopropyl β-D-thiogalactoside (IPTG) was added to the cultures of BL21+pEZY-Hb-ScAPX6 and BL21+pEZY-Hb to induce the expression of recombinant protein. The cultures were adjusted to OD_600_ = 0.6. Ten microliters from 10^−3^ (left side of the red line on the plate) to 10^−4^ (right side of the red line on the plate) dilutions were spotted onto LB plates without any supplement (CK) **(A)** or with NaCl (250, 500, and 750 mmol·L^−1^) **(B)**, PEG (15, 30, and 45%) **(C)** and CuCl_2_ (250, 500, and 750 μmol·L^−1^) **(D)**, respectively. NaCl, sodium chloride; PEG, polyethylene glycol; CuCl_2_, copper chloride.

It has been reported that the APX activity was up-regulated by abiotic stress, such as heavy metal, high salinity, drought, high temperature, and wounding (Shi et al., 2001). In this study, spot assay was performed to study the function of ScAPX6 in response to abiotic stress *in vivo*. The control (BL21+pEZY-Hb) and the gene-expressed cells (BL21+pEZY-Hb-*ScAPX6*) grew in LB plates containing NaCl, CuCl_2_, and PEG were performed (Figure 6). It had been recorded that the recombinant ScAPX6 cells showed a more rapid growth than the control in LB plates with PEG and CuCl_2_ supplement, but not with NaCl, suggesting that the overexpression of *ScAPX6* in *E. coli* could enhance its tolerance to PEG and Cu stress.

### Tissue-specific expression of *ScAPX6*

**Figure 7:**
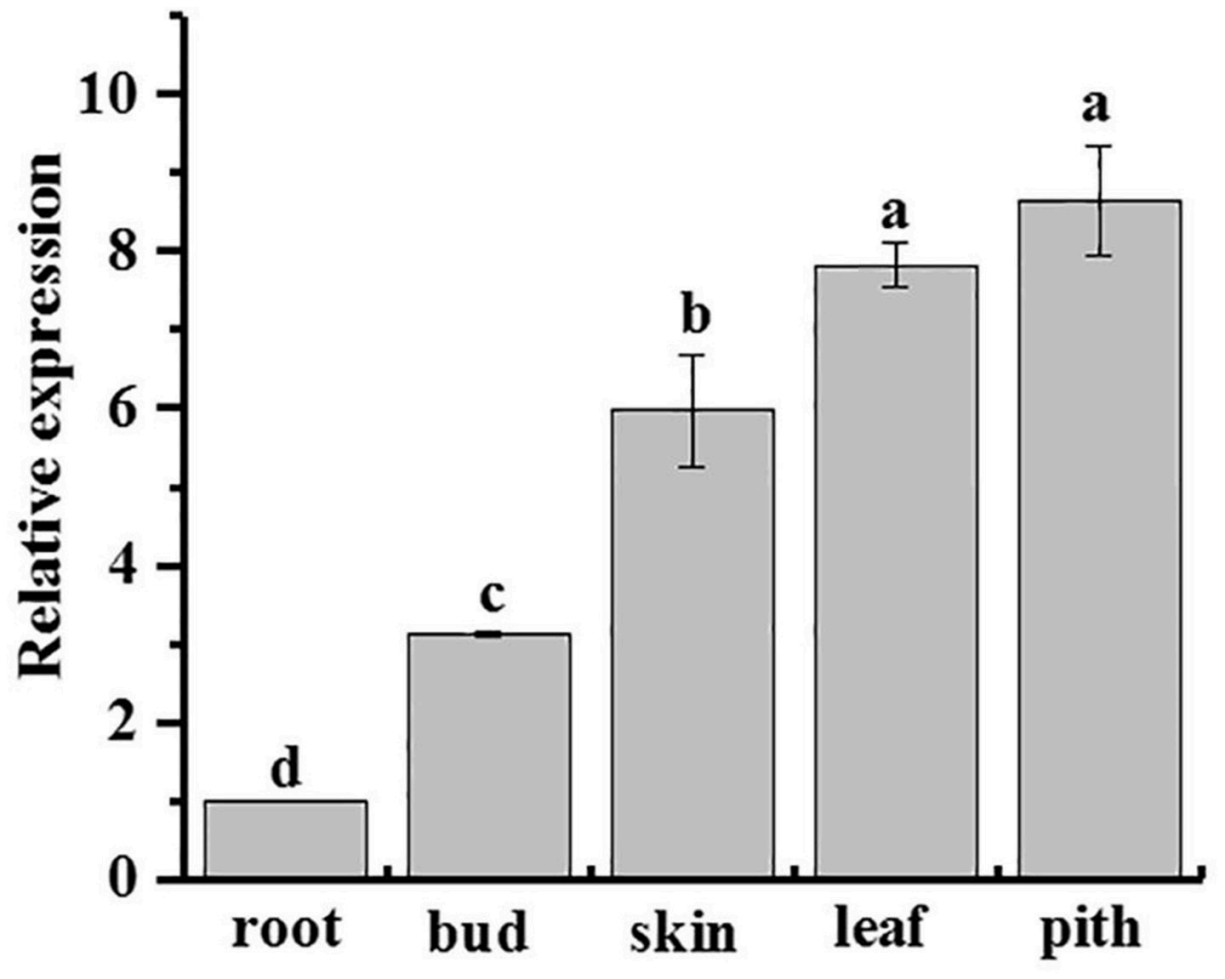
Tissue-specific expression analysis of *ScAPX6* in sugarcane. The error bars represented the standard error of each treating group (*n* = 3). Data were normalized to the *CAC* and *CUL* expression level. All data points were means ± SE (*n* = 3). Different lowercase letters indicate a significant difference, as determined by the Duncan's new multiple range test (*p* < 0.05).

qRT-PCR analysis showed that *ScAPX6* was constitutively expressed in all five kinds of sugarcane tissues, including root, bud, skin, leaf, and pith, but with different expression levels (Figure 7). *ScAPX6* showed the highest expression levels in the pith and leaf, and then on the skin, while the transcript in root was at the lowest level.

### Gene expression patterns of *ScAPX6* in response to abiotic stress

**Figure 8:**
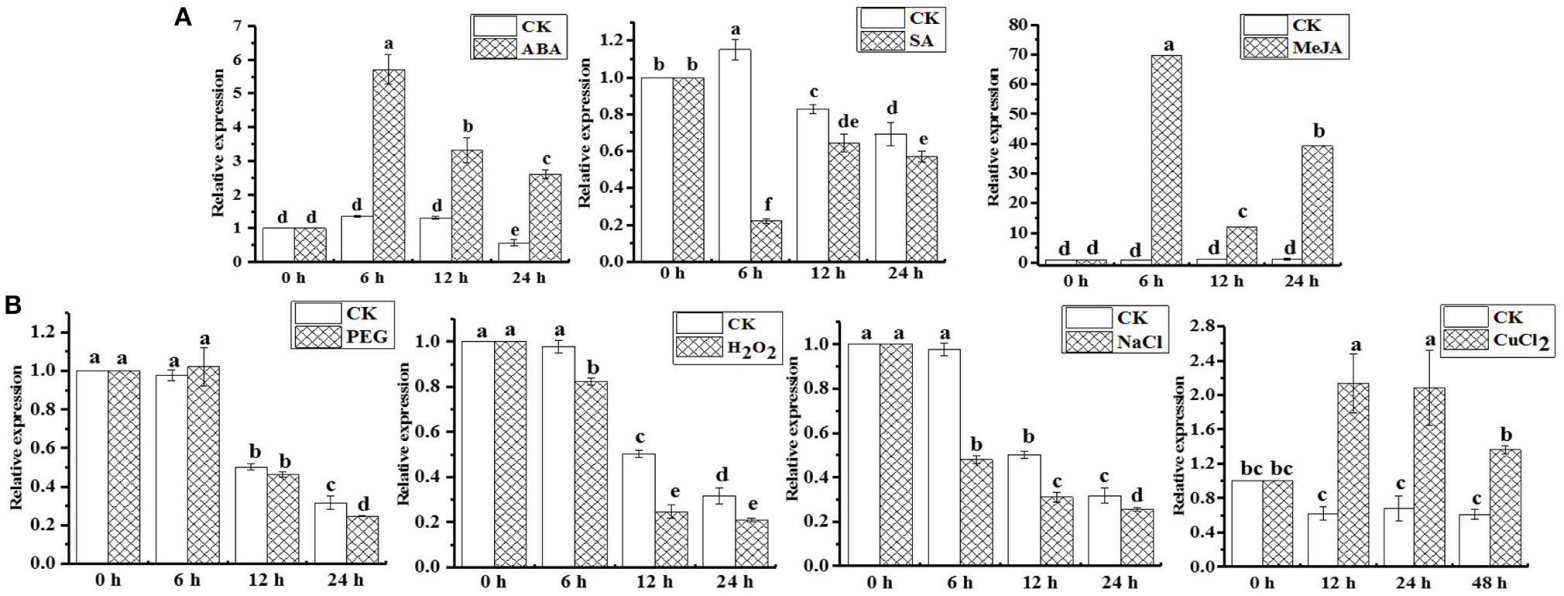
Gene expression patterns of *ScAPX6* in sugarcane under various plant hormones **(A)** and abiotic stresses **(B)**. Data were normalized to the *CAC* and *CUL* expression level. All data points were means ± SE (*n* = 3). Different lowercase letters indicate a significant difference, as determined by the Duncan's new multiple range test (*p* < 0.05). ABA, abscisic acid; SA, salicylic acid; MeJA, methyl jasmonate; PEG, polyethylene glycol; H_2_O_2_, hydrogen peroxide; NaCl, sodium chloride; CuCl_2_, copper chloride.

qRT-PCR analysis revealed that the *ScAPX6* gene exhibited different expression characteristics in response to ABA, MeJA, SA, H_2_O_2_, PEG, NaCl, and Cu stimuli (Figures 8A,B). As shown in Figure 8A, the transcripts of *ScAPX6* were remarkably up-regulated under the stresses of ABA and MeJA, and with the highest inducible expression levels at 6 h, which were 6.0- and 70.0-times higher than that of control, respectively. However, *ScAPX6* was down-regulated during the SA treatment and rapidly decreased at 6 h. Under the stress of PEG, the expression of *ScAPX6* showed no change at 6 h, and then decreased at 12 h. *ScAPX6* was down-regulated after the treatment of H_2_O_2_ and NaCl, but was up-regulated by the CuCl_2_. These results demonstrated that *ScAPX6* might positively respond to ABA, MeJA, and Cu stresses but negatively respond to SA, H_2_O_2_, PEG, and NaCl stresses.

### Transient overexpression of *ScAPX6* induces a defense response in *N. benthamiana*

**Figure 9:**
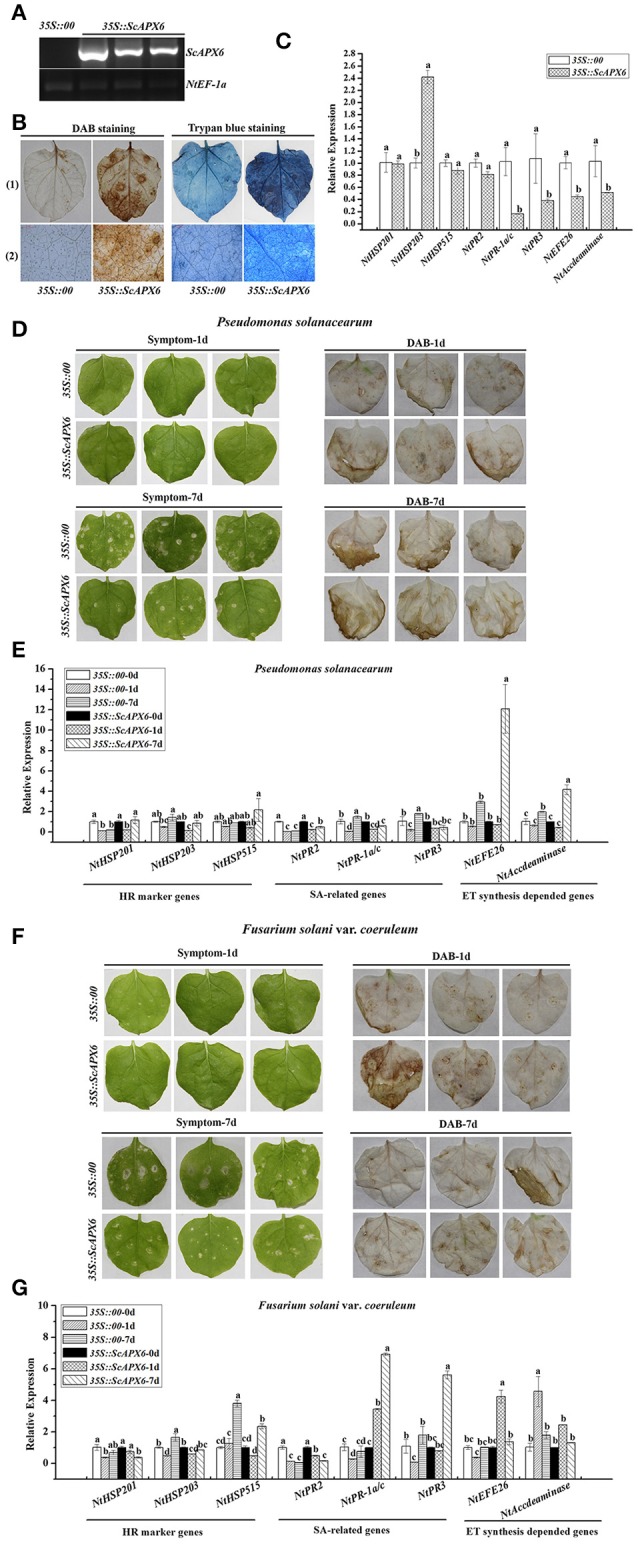
Transient overexpression of *ScAPX6* in *Nicotiana benthamiana* leaves. **(A)** RT-PCR analysis of *ScAPX6* in the *N. benthamiana* leaves after 1 d infiltration by *Agrobacterium* strain GV3101 carrying pEarleyGate 203-*ScAPX6* and the empty vector (*35S::00*). **(B)** DAB (3,3′-diaminobenzidinesolution) staining and trypan blue staining of *N. benthamiana* leaves at 48 h and 6 d after *Agrobacterium* strain infiltration, respectively, (1) represented a stereoscopic microscope and (2) represented a light microscope. **(C)** The transcripts of eight immunity-associated marker genes in the *N. benthamiana* leaves at 24 h after infiltration. **(D,F)** Disease symptoms and DAB staining results of *N. benthamiana* leaves by *P. solanacearum* and *F. solani* var. *coeruleum* infection after infiltration with *35S::00* (control) or *35S::ScAPX6*-containing *Agrobacterium* strain. Disease symptoms of infected leaves were observed at 1 and 7 d post-inoculation. **(E,G)** The transcripts of immunity-associated marker genes in the *N. benthamiana* leaves after inoculation with *P. solanacearum* or *F. solani* var. *coeruleum* for 1 and 7 d. *NtEF1-*α was used for normalization of the transcript levels. All data points were expressed as the mean ± SE (*n* = 3). Different lowercase letters indicate a significant difference, as determined by the Duncan's new multiple range test (*p* < 0.05). *NtHSR201, NtHSR203*, and *NtHSR515*, hypersensitive response marker genes; *NtPR2, NtPR-1a/c*, and *NtPR3*, a salicylic acid pathway-related gene; *NtEFE26* and *NtAccdeaminase*, the ethylene synthesis-dependent genes. Control, the *Agrobacterium* strain carrying *35S::00*.

After transient overexpression of *ScAPX6* in *N. benthamiana* leaves for 1 day (d), the transcripts of *ScAPX6* were detected by qRT-PCR (Figure 9A). As shown in Figures 9A,B darker DAB staining color and more intense trypan blue staining cells were observed in *ScAPX6* leaves than that in the control (*35S::00*) after infiltration for 2 and 6 d, respectively. The eight immunity-associated marker genes in *N. benthamiana* were induced by transient overexpression of *ScAPX6* (Figure 9C). The hypersensitive response (HR) marker genes, *NtHSR201* and *NtHSR203*, showed no change in transcript, while *NtHSR515* was up-regulated. The expression level of SA-responsive gene *NtPR2* remained unchanged, while *NtPR*-*1a/c* and *NtPR3* and two ethylene synthesis dependent genes, *NtEFE26* and *NtAccdeaminase*, were all down-regulated. Compared with the control leaves, *35S::ScAPX6* exhibited darker color reflecting high levels of H_2_O_2_ accumulation and intense hypersensitivity response.

To further investigate the response of *ScAPX6* to pathogen, two tobacco pathogens, *P. solanacearum* and *F. solani* var. *coeruleum*, were separately injected into *N. benthamiana* containing *35S::ScAPX6* or the control. After inoculation with *P. solanacearum*, no disease symptom was found between *35S::ScAPX6* and the control leaves for 1 d, while *35S::ScAPX6* exhibited darker color than the control at 1 d by DAB staining. With elongated treatment time, although the DAB staining showed no difference between *35S::ScAPX6* and the control leaves, the leaves in the control showed slight yellow phenomenon and necrotic spot, while the *35S::ScAPX6* only exhibited the faint wilting symptom after inoculation at 7 d (Figure 9D). After challenging with *P. solanacearum* for 1 and 7 d (Figure 9E), the expression levels of *NtHSR201, NtHSR203*, and *NtPR2*, were unchanged or down-regulated in the control and *35S::ScAPX6* leaves. The transcripts of *NtPR-1a/c* and *NtPR3* were significantly down-regulated at 1 d and increased at 7 d in the control leaves, but were down-regulated or remained unchanged in the *35S::ScAPX6* leaves. The expression levels of *NtHSR515* and *NtEFE26* were unchanged at 1 and 7 d and *NtAccdeaminase* was up-regulated at 7 d in the control leaves, while these three genes in the *35S::ScAPX6* leaves were all unchanged at 1 d and reached the peak values at 7 d after inoculation.

Likewise, for DAB staining, the *35S::ScAPX6* leaves showed darker color than the control after inoculation with *F. solani* var. *coeruleum* for 1 d and exhibited no difference at 7 d. No apparent disease symptom differences between *35S::ScAPX6* and the control were found at 1 d after inoculation (Figure 9F). Some symptoms, such as wilting, decay phenomenon and necrotic spot, were observed in the control leaves at 7 d, but not in the *35S::ScAPX6* leaves. After challenging with *F. solani* var. *coeruleum* for 1 and 7 d (Figure 9G), the expression levels of *NtHSR201* and *NtPR2* were unchanged or down-regulated in the control and *35S::ScAPX6* leaves. The transcripts of *NtAccdeaminase, NtHSR203*, and *NtHSR515*, were significantly up-regulated at 1 or 7 d in the control leaves, while stayed stable or showed a small rise in the *35S::ScAPX6* leaves. The transcripts of *NtPR-1a/c, NtPR3*, and *NtEFE26*, were down-regulated or remained unchanged in the control leaves, but were all significantly up-regulated in the *35S::ScAPX6* leaves.

## Discussion

Plant *APXs* are a multi-gene family (Shigeoka et al., 2002). Many plants *APXs* genes have been cloned and identified, including eight in *O. sativa* (Teixeira et al., 2005, 2006), four in *Vigna unguiculata* (D'Arcylameta et al., 2006) and *Spinacia oleracea*, respectively (Ishikawa et al., 1995, 1996, 1998), six in *Eucalyptus grandis* (Teixeira et al., 2005), seven in *Lycopersicon esculentum* (Najami et al., 2008), and nine in *A. thaliana* (Panchuk et al., 2002; Mittler et al., 2004; Narendra et al., 2006). In the present study, based on a putative *APX6* unigene sequence from our previous transcriptome data, a sugarcane *ScAPX6* gene (GenBank Acc. No. KT907352) was cloned (Figure S1), which was different from the other already reported sugarcane *APX* genes in NCBI (*ScAPX*: GenBank Acc. No. KJ7565501; *TAPX*: GenBank Acc. No. JQ958327; *APX*: GenBank Acc. No. KX235995), and shared only 19.79% identity at the amino acid sequence level. Najami et al. (2008) found that in *Solanum lycopersicum* three cytosolic *SlAPX* genes, *SlAPX1, SlAPX2*, and *SlAPX3* showed a high sequence identity (>90%). Teixeira et al. (2004) found that APX contained two isoforms, chloroplastic and nonchloroplastic isoforms. Furthermore, APx-R was found as a new heme-containing protein functionally associated with ascorbate peroxidase (Lazzarotto et al., 2011). In this study, ScAPX6 was clustered in chloroplastic isoforms (Figure 3), which was consistent with the study conducted by Teixeira et al. (2004). Subcellular localization of ScAPX6 in rice protoplast showed that ScAPX6::GFP was targeted at chloroplast (Figure 4), which was consistent with the result of bioinformatics predicted localization. Similar to other plant species, such as the APXs from *Cucumis melo* (Cheng et al., 2009) and *A. andraeanum* (Liu et al., 2013), ScAPX6 also contained a plant peroxidase like superfamily and the heme binding site and shared 94.29 and 82.93% similarities with the APX homologs from *S. italic* APX6 (XP_004973913.1) and *S. bicolor* APX6 (XP_002445876.1), suggesting that ScAPX6 belongs to a member of APX family.

*APX* plays an important role in response to biotic and abiotic stresses (Andréia et al., 2012). Cheng et al. (2009) observed that in *C. melo*, the gene expression level of *CmAPX* varied in different tissues, and with the highest expression in leaves and roots. Chen et al. (2011) found that *NuAPX* showed higher expression levels in leaf stalks than in root, due to the reasons that the tissues of the leaf stalks and leaf were rich in chloroplast and mitochondria, which was the leading source of reactive oxygen species (ROS) through the electron-transport chain of photosynthesis. In this study, *ScAPX6* was constitutively expressed in sugarcane tissues and with the highest expression in pith but the lowest in root (Figure 7).

For abiotic stress, Agarwal et al. (2005) have found that the enzyme activities of APX, SOD, and CAT in wheat seedlings could be increased by 500 mmol·L^−1^ ABA treatment. It was shown previously that in *H. vulgare*, the transcript level of *HvAPX1* was remarkably up-regulated by the treatments of ABA and NaCl (Shi et al., 2001). In *Brassica oleracea* var. *italica*, Jiang et al. (2012) have found that the expression of *BoAPX2* increased after H_2_O_2_, SA, and NaCl treatments. Previous research on sugarcane showed that the transcripts of *ScAPX* increased under the treatment of ABA, MeJA, SA, H_2_O_2_, PEG, and NaCl (Wang Z. Q. et al., 2015) In this study, the expression of *ScAPX6* was also up-regulated by both ABA and MeJA, but down-regulated by SA and H_2_O_2_ treatments (Figure 8A). As reported, the expression of *TAPX* gene in sugarcane was significantly induced by NaCl and PEG stresses (Wang S. et al., 2015). Previous investigations have identified the transcripts of *OsAPX7* and *OsAPX8*, which were separately down regulated by 300 mmol·L^−1^ NaCl in rice roots and leaves (Hong and Kao, 2007; Yamane et al., 2010). Similarly, in this study, down regulation of the transcripts of *ScAPX6* were noted with NaCl and PEG treatment (Figure 8B), which was consistent with the results of the spot assay that the recombinant protein of ScAPX6 expressed in *E. coli* BL21 did not show better growth than the control under both two treatments (Figure 6). Previous reports have provided evidence that over-expressed plant stress tolerance genes in *E. coli* cells could enhance their growth under abiotic stress (Gupta et al., 2010; Guo et al., 2013). For example, Su et al. (2014b) have tested a chitinase gene *ScChi* in *E. coli*, which showed better growth under NaCl, Cu, CdCl_2_, and ZnSO_4_ treatments. Duan et al. (2006) have indicated that the transgenic *O. sativa* with *HvAPX1* gene was more tolerant to cadmium stress when compared with the wild type. In this study, the transcript of *ScAPX6* was also up-regulated by the treatment of Cu (Figure 8B), which was in line with the results that the recombinant protein of ScAPX6 expressed in *E. coli* BL21 resulted in a better growth under Cu stress (Figure 6). Therefore, it was predicted that *ScAPX6* could be helpful for the tolerance of sugarcane to Cu. These findings suggested *ScAPX6* might be a positive response to ABA, MeJA, and Cu stresses, while showed the negative response to SA, H_2_O_2_, PEG, and NaCl stresses. However, what should be pointed out here is that, we only use treatment with some hormones, such as SA, ABA, and MeJA with one concentration, and we cannot directly link effect of our treatment with specific hormone pathway because the specific concentration of the hormones was not determined. These points need to be considered in future.

Previous studies have revealed that the overexpression of *tApx* gene in tobacco enhanced tolerance to chilling, methylvioiogen, and high-intensity light (Yabuta et al., 2002). Transgenic potato with simultaneous overexpression of *APX*, choline oxidase (codA), and *SOD*, increased tolerance of SSAC plants and lower levels of H_2_O_2_ under methylvioiogen, drought and salt-mediated oxidative stresses (Ahmad et al., 2010). Investigations have revealed that cell death could induce R gene expression, ion fluxes, stimulation of ROS, and defense-related hormones, which can efficiently restrict pathogen growth and development (Li et al., 2010; Melech-Bonfil and Sessa, 2010; Du et al., 2012). Thordal-Christensen et al. (1997) proved that DAB-uptake method can serve H_2_O_2_ detection at a subcellular level. Although the DAB reactions reflect increases in local H_2_O_2_, this method has been adapted to many other plant species for in situ detection of H_2_O_2_. Thordal-Christensen et al. (1997) speculated that this could be related either to the fact that the optimal pH (5.5–6.0) for DAB precipitation coincides with the expected pH level in the leaf, or to the strong polymerization observed in the plant tissue. Lai et al. (2013) found that *35S::BrERF11* transgenic tobacco plants showed significantly increased HR and H_2_O_2_ accumulation compared with wild-type plants according to trypan blue and DAB staining. In the present study, a darker DAB staining color was found after overexpression of *ScAPX6* in *N. benthamiana* leaves and after inoculation with *P. solanacearum* and *F. solani* var. *coeruleum* at 1 d compared with that in the leaves of control (Figures 9B,D,F), which was indicative of the accumulation of H_2_O_2_ and resulted in intense hypersensitivity response, but with elongated treatment time, the DAB staining color became lighter, which was consistent with the fact that APX can rapidly scavenge H_2_O_2_ in the plant by the ASA-GSH cycle (Shigeoka et al., 2002). However, since the DAB buffer is far from real physiological conditions, whether itself results in the accumulation of H_2_O_2_ remains an open question. Together, the antimicrobial action against the tobacco pathogens after overexpression of *ScAPX6* in *N. benthamiana*, suggesting that *ScAPX6* may enhance the resistance to *P. solanacearum* and *F. solani* var. *coeruleum*.

## Conclusions

In this study, a novel ascorbate peroxidase gene, *ScAPX6* (GenBank Acc. No. KT907352), was isolated and characterized. The cDNA of *ScAPX6* gene was 1,086 bp long with a complete 1,002 bp ORF, encoding 333 amino acids. Subcellular localization revealed that ScAPX6 was targeted in chloroplast. After inducing by IPTG, the accumulation protein of pEZYHb-*ScAPX6* at 55 KDa led to a better growth of *E. coli* BL21 under Cu stress. *ScAPX6* was constitutively expressed in sugarcane tissues. Besides, *ScAPX6* showed positive response to ABA, MeJA, and Cu stresses, but negative response to the stresses of SA, H_2_O_2_, PEG, and NaCl. The overexpression of *ScAPX6* in *N. benthamiana* leaves showed positive response against the attack of *P. solanacearum* and *F. solani* var. *coeruleum*. These results suggested that *ScAPX6* plays an important role in the HR or immunity of sugarcane.

## Author contributions

FL, YS, and YQ: Conceived, designed, and initiated the project; NH, HL, and SG: Prepared materials; FL, NH, LW, TS, WA, and JG: Performed experiments and contributed to data analysis and validation; FL and YS: Drafted the manuscript; LX, KM, YS, and YQ: Revised the manuscript. All authors read and approved the final manuscript.

### Conflict of interest statement

The authors declare that the research was conducted in the absence of any commercial or financial relationships that could be construed as a potential conflict of interest.

## Supplementary material

Figure S1 Amplification of *ScAPX6* gene in sugarcane. M: DNA marker 2,000 bp; 1: RT-PCR product.
