# Supplementary figures and images for: A Novel L-ascorbate Peroxidase 6 Gene, ScAPX6, Plays an Important Role in the Regulation of Response to Biotic and Abiotic Stresses in Sugarcane
Source: Front Plant Sci. 2018 Jan 17;8:2262. doi: 10.3389/fpls.2017.02262 (PMC5776131; doi:10.3389/fpls.2017.02262)

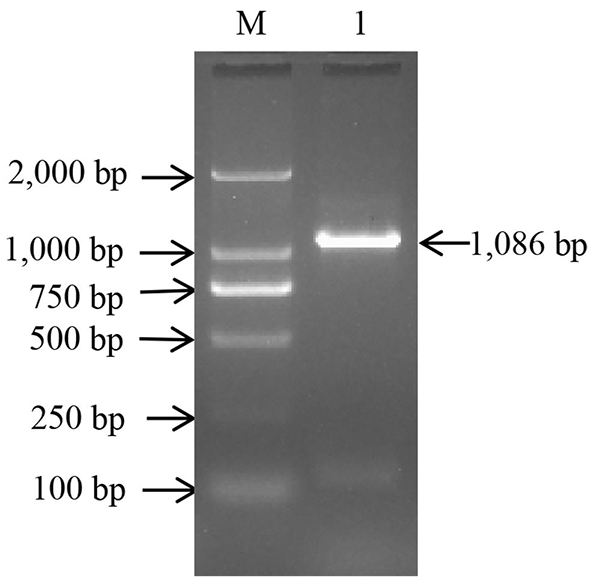

Supplement: Figure S1 — Amplification of ScAPX6 gene in sugarcane. M: DNA marker 2,000 bp; 1: RT-PCR product. [file Image1.TIF]
